# 5-Amino-2,4,6-triiodo­isophthalic acid–4,4′-bipyridine *N*,*N*′-dioxide–water (1/1/1)

**DOI:** 10.1107/S1600536811007276

**Published:** 2011-03-05

**Authors:** Kou-Lin Zhang, Jin-Bo Zhang, Seik Weng Ng

**Affiliations:** a College of Chemistry and Chemical Engineering, Yangzhou University, Yangzhou 225002, People’s Republic of China; b Department of Chemistry, University of Malaya, 50603 Kuala Lumpur, Malaysia

## Abstract

The aromatic rings of the *N*,*N*′-dioxide molecule in the title compound, C_8_H_4_NI_3_O_4_·C_10_H_8_N_2_O_2_·H_2_O, are twisted by 14.0 (2)°. The –CO_2_H substituents of the 5-amino-2,4,6-triiodo­isophthalic acid are twisted by 83.0 (2) and 86.5 (2)° out of the plane of the aromatic ring. In the crystal, the three components are linked by O—H⋯O hydrogen bonds into a three-dimensional network. An N—H⋯O inter­action also occurs. One of the amino H atom is not involved in hydrogen bonding.

## Related literature

For the structure of the monohydrated carb­oxy­lic acid, see: Beck & Sheldrick (2008 ▶). For the 4,4′-bipyridinium 5-amino-2,4,6-triiodo­isophthalate co-crystal of carb­oxy­lic acid, see: Zhang *et al.* (2010 ▶).
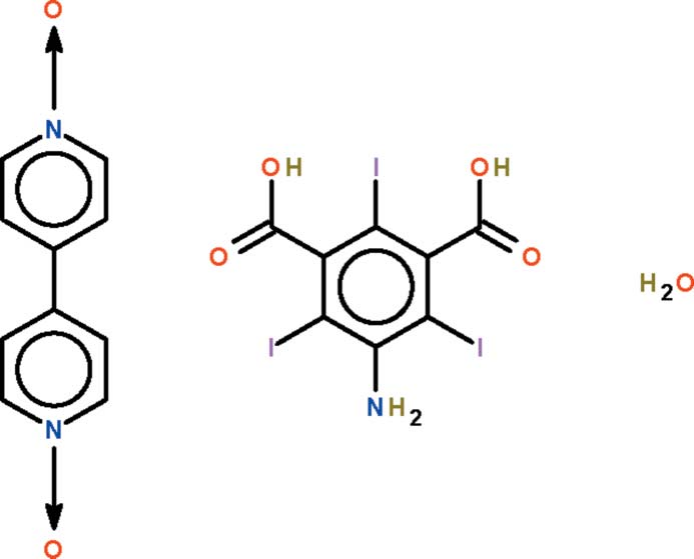


## Experimental

### 

#### Crystal data

C_8_H_4_NI_3_O_4_·C_10_H_8_N_2_O_2_·H_2_O
*M* *_r_* = 765.02
Monoclinic, 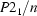

*a* = 7.5000 (2) Å
*b* = 17.0808 (4) Å
*c* = 16.523 (3) Å
β = 94.349 (2)°
*V* = 2110.6 (4) Å^3^
*Z* = 4
Mo *K*α radiation
μ = 4.49 mm^−1^
*T* = 100 K
0.20 × 0.05 × 0.05 mm

#### Data collection

Agilent SuperNova Dual diffractometer with an Atlas detector
Absorption correction: multi-scan (*CrysAlis PRO*; Agilent, 2010 ▶) *T*_min_ = 0.467, *T*_max_ = 0.807
10861 measured reflections
4660 independent reflections
4112 reflections with *I* > 2σ(*I*)
*R*_int_ = 0.032

#### Refinement

*R*[*F* ^2^ > 2σ(*F* ^2^)] = 0.029
*wR*(*F* ^2^) = 0.068
*S* = 1.04
4660 reflections
304 parameters
6 restraints
H atoms treated by a mixture of independent and constrained refinement
Δρ_max_ = 0.73 e Å^−3^
Δρ_min_ = −0.97 e Å^−3^

### 

Data collection: *CrysAlis PRO* (Agilent, 2010 ▶); cell refinement: *CrysAlis PRO*; data reduction: *CrysAlis PRO*; program(s) used to solve structure: *SHELXS97* (Sheldrick, 2008 ▶); program(s) used to refine structure: *SHELXL97* (Sheldrick, 2008 ▶); molecular graphics: *X-SEED* (Barbour, 2001 ▶); software used to prepare material for publication: *publCIF* (Westrip, 2010 ▶).

## Supplementary Material

Crystal structure: contains datablocks global, I. DOI: 10.1107/S1600536811007276/bt5482sup1.cif

Structure factors: contains datablocks I. DOI: 10.1107/S1600536811007276/bt5482Isup2.hkl

Additional supplementary materials:  crystallographic information; 3D view; checkCIF report

## Figures and Tables

**Table 1:** Hydrogen-bond geometry (Å, °)

*D*—H⋯*A*	*D*—H	H⋯*A*	*D*⋯*A*	*D*—H⋯*A*
O1—H1⋯O5^i^	0.84 (3)	1.64 (3)	2.478 (4)	174 (6)
O3—H3⋯O6^ii^	0.84 (3)	1.63 (3)	2.465 (4)	170 (7)
O1*W*—H1w1⋯O2	0.84 (3)	2.30 (3)	3.073 (4)	154 (4)
O1*W*—H1w2⋯O5^iii^	0.84 (3)	2.12 (3)	2.945 (4)	167 (5)
N1—H11⋯O1w^iv^	0.88 (3)	2.20 (3)	2.906 (5)	138 (4)

Symmetry codes: (i) 
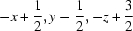
; (ii) 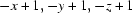; (iii) 
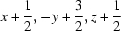
; (iv) 
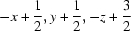
.

**Fig. 1.**
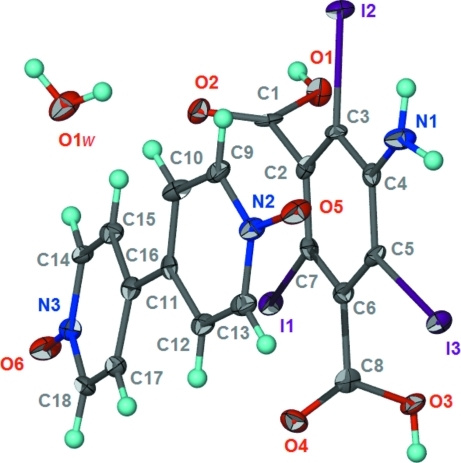
Anisotropic displacement ellipsoid plot (Barbour, 2001) of C10H8N2O2.C8H4NI3O4.H2O at the 70% probability level; hydrogen atoms are drawn as spheres of arbitrary radius.

## supplementary crystallographic information

### Comment

The attempt at synthesizing the 4,4'-bipyridine adduct of cadmium 5-amino-2,4,6-triiodoisophthalate gave instead a co-crystal having a monoprotonated 4,4'-bipyridinium 5-amino-2,4,6-triiodoisophthalate as one component and a carboxylic acid as the other (Zhang *et al.*, 2010). Replacing the metal ion by a zinc ion, and with 4,4'-bipyridine *N*,*N*'-dioxide in place of 4,4'-bipyridine, gave instead the title monohydrated neutral co-crystal, C_10_H_8_N_2_O_2_^.^C_8_H_4_NI_2_O_4_^.^H_2_O (Scheme I, Fig. 1). In the *N*-heterocycle, the rings are twisted by 14.0 (2) °. In the carboxylic acid, the –CO_2_H substituents are nearly perpendicular to the aromatic ring. The three components are linked by O–H···O hydrogen bonds into a layer structure (Table 1).

### Experimental

Zinc nitrate hexahydrate (58 mg, 0.2 mmol), 5-amino-2,4,6-triiodoisophthalic acid (59 mg, 0.1 mmol), 4,4'-bipyridine *N*,*N*'-dioxide (56 mg, 0.1 mmol), sodium hydroxide (4 mg, 0.1 mmol) and water (6 ml) were heated ain a 16-ml, Teflon-lined Parr bomb. The bomb was heated at 343 K for 3 days. Greenish-yellow crystals were isolated from the cool mixture.

### Refinement

Carbon-bound H-atoms were placed in calculated positions [C—H 0.95 Å, *U*_iso_(H) 1.2 to 1.5*U*_eq_(C)] and were included in the refinement in the riding model approximation.

The amino and water H-atoms were located in a difference Fourier map, and were refined with distance restraints of N–H = 0.88±0.01, O–H = 0.84±0.01 Å.

### Crystal data

**Table:**

C_8_H_4_NI_3_O_4_·C_10_H_8_N_2_O_2_·H_2_O	*F*(000) = 1432
*M**_r_* = 765.02	*D*_x_ = 2.408 Mg m^−^^3^
Monoclinic, *P*2_1_/*n*	Mo *K*α radiation, λ = 0.71073 Å
Hall symbol: -P 2yn	Cell parameters from 5995 reflections
*a* = 7.5000 (2) Å	θ = 2.4–29.2°
*b* = 17.0808 (4) Å	µ = 4.49 mm^−^^1^
*c* = 16.523 (3) Å	*T* = 100 K
β = 94.349 (2)°	Prism, yellow
*V* = 2110.6 (4) Å^3^	0.20 × 0.05 × 0.05 mm
*Z* = 4	

### Data collection

**Table:**

Agilent SuperNova Dual diffractometer with an Atlas detector	4660 independent reflections
Radiation source: SuperNova (Mo) X-ray Source	4112 reflections with *I* > 2σ(*I*)
Mirror	*R*_int_ = 0.032
Detector resolution: 10.4041 pixels mm^-1^	θ_max_ = 27.5°, θ_min_ = 2.4°
ω scans	*h* = −9→9
Absorption correction: multi-scan (*CrysAlis PRO*; Agilent, 2010)	*k* = −16→21
*T*_min_ = 0.467, *T*_max_ = 0.807	*l* = −21→21
10861 measured reflections	

### Refinement

**Table:**

Refinement on *F*^2^	Primary atom site location: structure-invariant direct methods
Least-squares matrix: full	Secondary atom site location: difference Fourier map
*R*[*F*^2^ > 2σ(*F*^2^)] = 0.029	Hydrogen site location: inferred from neighbouring sites
*wR*(*F*^2^) = 0.068	H atoms treated by a mixture of independent and constrained refinement
*S* = 1.04	*w* = 1/[σ^2^(*F*_o_^2^) + (0.0304*P*)^2^] where *P* = (*F*_o_^2^ + 2*F*_c_^2^)/3
4660 reflections	(Δ/σ)_max_ = 0.001
304 parameters	Δρ_max_ = 0.73 e Å^−^^3^
6 restraints	Δρ_min_ = −0.97 e Å^−^^3^

### Fractional atomic coordinates and isotropic or equivalent isotropic displacement parameters (Å^2^)

**Table:**

	*x*	*y*	*z*	*U*_iso_*/*U*_eq_	
I1	0.32943 (3)	0.507910 (15)	0.632563 (14)	0.01280 (8)	
I2	0.05617 (3)	0.779896 (16)	0.826265 (14)	0.01348 (8)	
I3	0.05801 (3)	0.802046 (16)	0.463723 (14)	0.01291 (8)	
O1	0.0967 (4)	0.57411 (17)	0.81306 (15)	0.0145 (6)	
O2	0.3866 (4)	0.60594 (16)	0.82653 (15)	0.0139 (6)	
O3	0.1102 (4)	0.59971 (17)	0.45040 (15)	0.0140 (6)	
O4	0.3967 (4)	0.63963 (17)	0.46318 (15)	0.0158 (6)	
O5	0.3523 (4)	0.98110 (17)	0.57244 (15)	0.0171 (6)	
O6	0.8488 (4)	0.46621 (17)	0.67957 (15)	0.0165 (6)	
O1W	0.6478 (5)	0.49085 (19)	0.91589 (18)	0.0215 (7)	
N1	0.0344 (5)	0.8521 (2)	0.64989 (18)	0.0164 (8)	
N2	0.4406 (4)	0.9148 (2)	0.59092 (18)	0.0134 (7)	
N3	0.8019 (4)	0.5409 (2)	0.66729 (18)	0.0126 (7)	
C1	0.2386 (5)	0.6091 (2)	0.7914 (2)	0.0111 (8)	
C2	0.2034 (5)	0.6574 (2)	0.7142 (2)	0.0112 (8)	
C3	0.1342 (5)	0.7325 (2)	0.7169 (2)	0.0099 (8)	
C4	0.0991 (5)	0.7782 (2)	0.6459 (2)	0.0109 (8)	
C5	0.1361 (5)	0.7429 (2)	0.5724 (2)	0.0105 (8)	
C6	0.2090 (5)	0.6682 (2)	0.5683 (2)	0.0105 (8)	
C7	0.2412 (5)	0.6246 (2)	0.6397 (2)	0.0098 (8)	
C8	0.2487 (5)	0.6338 (2)	0.4873 (2)	0.0119 (8)	
C9	0.4693 (5)	0.8909 (3)	0.6689 (2)	0.0145 (9)	
H9	0.4314	0.9225	0.7116	0.017*	
C10	0.5527 (6)	0.8211 (2)	0.6861 (2)	0.0149 (9)	
H10	0.5746	0.8057	0.7412	0.018*	
C11	0.6067 (5)	0.7718 (2)	0.6253 (2)	0.0117 (8)	
C12	0.5799 (5)	0.8009 (2)	0.5456 (2)	0.0129 (8)	
H12A	0.6200	0.7712	0.5019	0.016*	
C13	0.4974 (5)	0.8712 (2)	0.5301 (2)	0.0144 (8)	
H13	0.4798	0.8894	0.4757	0.017*	
C14	0.7350 (5)	0.5812 (2)	0.7277 (2)	0.0123 (8)	
H14	0.7275	0.5570	0.7791	0.015*	
C15	0.6778 (5)	0.6564 (2)	0.7165 (2)	0.0130 (8)	
H15	0.6333	0.6841	0.7606	0.016*	
C16	0.6833 (5)	0.6936 (2)	0.6412 (2)	0.0133 (8)	
C17	0.7574 (5)	0.6498 (2)	0.5799 (2)	0.0127 (8)	
H17	0.7661	0.6726	0.5279	0.015*	
C18	0.8169 (5)	0.5751 (2)	0.5935 (2)	0.0130 (8)	
H18	0.8688	0.5469	0.5516	0.016*	
H1	0.121 (7)	0.543 (2)	0.852 (2)	0.038 (16)*	
H3	0.130 (8)	0.582 (3)	0.4044 (17)	0.06 (2)*	
H1W1	0.563 (5)	0.523 (2)	0.907 (3)	0.031 (15)*	
H1W2	0.694 (6)	0.505 (3)	0.9614 (14)	0.026 (14)*	
H11	−0.016 (6)	0.877 (2)	0.6082 (18)	0.023 (13)*	
H12	−0.008 (6)	0.870 (3)	0.6942 (16)	0.029 (13)*	

### Atomic displacement parameters (Å^2^)

**Table:**

	*U*^11^	*U*^22^	*U*^33^	*U*^12^	*U*^13^	*U*^23^
I1	0.01590 (14)	0.00943 (15)	0.01275 (13)	0.00111 (10)	−0.00109 (10)	−0.00108 (10)
I2	0.01938 (15)	0.01238 (15)	0.00890 (12)	0.00079 (11)	0.00256 (10)	−0.00099 (10)
I3	0.01642 (14)	0.01286 (15)	0.00930 (12)	0.00009 (11)	−0.00009 (10)	0.00293 (10)
O1	0.0152 (15)	0.0146 (16)	0.0137 (13)	−0.0024 (12)	0.0007 (11)	0.0074 (12)
O2	0.0138 (15)	0.0163 (16)	0.0112 (12)	0.0002 (12)	−0.0024 (11)	0.0021 (12)
O3	0.0166 (15)	0.0146 (16)	0.0105 (13)	−0.0011 (12)	−0.0015 (11)	−0.0046 (12)
O4	0.0153 (15)	0.0192 (17)	0.0135 (13)	0.0012 (13)	0.0053 (11)	0.0015 (12)
O5	0.0252 (17)	0.0114 (16)	0.0140 (13)	0.0066 (13)	−0.0029 (12)	−0.0030 (12)
O6	0.0256 (17)	0.0105 (15)	0.0131 (13)	0.0048 (13)	0.0001 (12)	−0.0012 (12)
O1W	0.0281 (19)	0.0166 (18)	0.0185 (15)	0.0011 (15)	−0.0061 (14)	−0.0044 (13)
N1	0.025 (2)	0.014 (2)	0.0102 (16)	0.0045 (16)	0.0026 (15)	0.0014 (15)
N2	0.0162 (18)	0.0109 (19)	0.0130 (15)	−0.0011 (14)	0.0001 (13)	−0.0032 (14)
N3	0.0123 (17)	0.0139 (19)	0.0117 (15)	0.0012 (14)	0.0007 (13)	−0.0001 (14)
C1	0.020 (2)	0.009 (2)	0.0052 (16)	−0.0004 (17)	0.0029 (15)	−0.0027 (15)
C2	0.0104 (19)	0.012 (2)	0.0104 (17)	−0.0018 (16)	−0.0024 (14)	−0.0001 (15)
C3	0.0112 (19)	0.011 (2)	0.0080 (16)	−0.0031 (16)	0.0021 (14)	−0.0052 (15)
C4	0.0080 (19)	0.011 (2)	0.0134 (18)	−0.0018 (16)	−0.0006 (14)	−0.0013 (15)
C5	0.013 (2)	0.013 (2)	0.0055 (16)	−0.0021 (16)	−0.0017 (14)	0.0029 (15)
C6	0.0103 (19)	0.011 (2)	0.0102 (17)	−0.0017 (16)	0.0004 (14)	0.0003 (15)
C7	0.0083 (19)	0.008 (2)	0.0134 (17)	0.0020 (15)	0.0000 (14)	0.0006 (15)
C8	0.018 (2)	0.008 (2)	0.0098 (17)	0.0039 (17)	−0.0009 (15)	0.0059 (15)
C9	0.016 (2)	0.016 (2)	0.0114 (17)	0.0011 (17)	−0.0027 (15)	−0.0044 (16)
C10	0.021 (2)	0.015 (2)	0.0082 (17)	−0.0017 (18)	0.0001 (15)	−0.0001 (16)
C11	0.0058 (18)	0.015 (2)	0.0142 (18)	−0.0047 (16)	0.0001 (14)	−0.0012 (16)
C12	0.013 (2)	0.013 (2)	0.0125 (18)	−0.0043 (17)	0.0006 (15)	−0.0046 (16)
C13	0.016 (2)	0.015 (2)	0.0121 (17)	−0.0024 (18)	0.0012 (15)	−0.0024 (16)
C14	0.0111 (19)	0.017 (2)	0.0082 (17)	−0.0022 (17)	0.0008 (14)	−0.0036 (16)
C15	0.013 (2)	0.013 (2)	0.0124 (17)	−0.0029 (17)	0.0011 (15)	−0.0031 (16)
C16	0.010 (2)	0.017 (2)	0.0131 (18)	−0.0032 (17)	−0.0002 (15)	−0.0033 (16)
C17	0.0102 (19)	0.017 (2)	0.0106 (17)	−0.0027 (17)	0.0017 (14)	−0.0011 (16)
C18	0.0089 (19)	0.019 (2)	0.0117 (17)	0.0011 (17)	0.0028 (14)	−0.0029 (16)

### Geometric parameters (Å, °)

**Table:**

I1—C7	2.106 (4)	C3—C4	1.417 (5)
I2—C3	2.103 (4)	C4—C5	1.402 (5)
I3—C5	2.104 (4)	C5—C6	1.392 (6)
O1—C1	1.294 (5)	C6—C7	1.400 (5)
O1—H1	0.84 (3)	C6—C8	1.512 (5)
O2—C1	1.214 (4)	C9—C10	1.365 (6)
O3—C8	1.302 (5)	C9—H9	0.9500
O3—H3	0.84 (3)	C10—C11	1.394 (6)
O4—C8	1.212 (5)	C10—H10	0.9500
O5—N2	1.335 (4)	C11—C12	1.407 (5)
O6—N3	1.335 (4)	C11—C16	1.469 (6)
O1W—H1W1	0.84 (3)	C12—C13	1.366 (6)
O1W—H1W2	0.84 (3)	C12—H12A	0.9500
N1—C4	1.356 (5)	C13—H13	0.9500
N1—H11	0.88 (3)	C14—C15	1.362 (6)
N1—H12	0.88 (3)	C14—H14	0.9500
N2—C13	1.347 (5)	C15—C16	1.402 (5)
N2—C9	1.354 (5)	C15—H15	0.9500
N3—C14	1.341 (5)	C16—C17	1.408 (5)
N3—C18	1.364 (5)	C17—C18	1.365 (6)
C1—C2	1.525 (5)	C17—H17	0.9500
C2—C3	1.386 (5)	C18—H18	0.9500
C2—C7	1.401 (5)		
			
C1—O1—H1	111 (4)	O4—C8—O3	126.9 (3)
C8—O3—H3	113 (4)	O4—C8—C6	120.4 (4)
H1W1—O1W—H1W2	103 (5)	O3—C8—C6	112.6 (3)
C4—N1—H11	124 (3)	N2—C9—C10	120.0 (4)
C4—N1—H12	122 (3)	N2—C9—H9	120.0
H11—N1—H12	108 (4)	C10—C9—H9	120.0
O5—N2—C13	118.6 (3)	C9—C10—C11	122.0 (4)
O5—N2—C9	121.0 (3)	C9—C10—H10	119.0
C13—N2—C9	120.4 (4)	C11—C10—H10	119.0
O6—N3—C14	119.2 (3)	C10—C11—C12	115.6 (4)
O6—N3—C18	120.4 (3)	C10—C11—C16	123.4 (3)
C14—N3—C18	120.4 (4)	C12—C11—C16	121.0 (3)
O2—C1—O1	126.0 (3)	C13—C12—C11	121.2 (4)
O2—C1—C2	121.2 (3)	C13—C12—H12A	119.4
O1—C1—C2	112.8 (3)	C11—C12—H12A	119.4
C3—C2—C7	120.0 (3)	N2—C13—C12	120.7 (4)
C3—C2—C1	121.0 (3)	N2—C13—H13	119.7
C7—C2—C1	119.0 (4)	C12—C13—H13	119.7
C2—C3—C4	121.9 (3)	N3—C14—C15	121.0 (4)
C2—C3—I2	120.9 (3)	N3—C14—H14	119.5
C4—C3—I2	117.0 (3)	C15—C14—H14	119.5
N1—C4—C5	122.5 (3)	C14—C15—C16	121.2 (4)
N1—C4—C3	121.1 (3)	C14—C15—H15	119.4
C5—C4—C3	116.4 (4)	C16—C15—H15	119.4
C6—C5—C4	122.7 (3)	C15—C16—C17	116.0 (4)
C6—C5—I3	119.0 (2)	C15—C16—C11	122.2 (4)
C4—C5—I3	118.2 (3)	C17—C16—C11	121.7 (3)
C5—C6—C7	119.4 (3)	C18—C17—C16	121.4 (4)
C5—C6—C8	120.2 (3)	C18—C17—H17	119.3
C7—C6—C8	120.4 (3)	C16—C17—H17	119.3
C6—C7—C2	119.6 (4)	N3—C18—C17	119.9 (3)
C6—C7—I1	119.4 (3)	N3—C18—H18	120.0
C2—C7—I1	120.9 (3)	C17—C18—H18	120.0
			
O2—C1—C2—C3	−97.1 (5)	C5—C6—C8—O4	94.5 (5)
O1—C1—C2—C3	82.9 (5)	C7—C6—C8—O4	−87.0 (5)
O2—C1—C2—C7	83.2 (5)	C5—C6—C8—O3	−85.4 (5)
O1—C1—C2—C7	−96.8 (4)	C7—C6—C8—O3	93.1 (4)
C7—C2—C3—C4	0.2 (6)	O5—N2—C9—C10	177.0 (4)
C1—C2—C3—C4	−179.5 (4)	C13—N2—C9—C10	−1.5 (6)
C7—C2—C3—I2	174.9 (3)	N2—C9—C10—C11	−1.6 (6)
C1—C2—C3—I2	−4.8 (5)	C9—C10—C11—C12	3.9 (6)
C2—C3—C4—N1	−178.8 (4)	C9—C10—C11—C16	−174.8 (4)
I2—C3—C4—N1	6.3 (5)	C10—C11—C12—C13	−3.4 (6)
C2—C3—C4—C5	0.8 (6)	C16—C11—C12—C13	175.4 (4)
I2—C3—C4—C5	−174.1 (3)	O5—N2—C13—C12	−176.5 (3)
N1—C4—C5—C6	177.4 (4)	C9—N2—C13—C12	2.0 (6)
C3—C4—C5—C6	−2.1 (6)	C11—C12—C13—N2	0.6 (6)
N1—C4—C5—I3	−7.7 (5)	O6—N3—C14—C15	−176.9 (3)
C3—C4—C5—I3	172.7 (3)	C18—N3—C14—C15	1.2 (6)
C4—C5—C6—C7	2.5 (6)	N3—C14—C15—C16	1.3 (6)
I3—C5—C6—C7	−172.3 (3)	C14—C15—C16—C17	−2.4 (6)
C4—C5—C6—C8	−179.0 (4)	C14—C15—C16—C11	174.0 (4)
I3—C5—C6—C8	6.2 (5)	C10—C11—C16—C15	12.1 (6)
C5—C6—C7—C2	−1.4 (6)	C12—C11—C16—C15	−166.5 (4)
C8—C6—C7—C2	−179.9 (4)	C10—C11—C16—C17	−171.6 (4)
C5—C6—C7—I1	174.5 (3)	C12—C11—C16—C17	9.7 (6)
C8—C6—C7—I1	−4.0 (5)	C15—C16—C17—C18	1.1 (6)
C3—C2—C7—C6	0.1 (6)	C11—C16—C17—C18	−175.4 (4)
C1—C2—C7—C6	179.8 (3)	O6—N3—C18—C17	175.6 (3)
C3—C2—C7—I1	−175.7 (3)	C14—N3—C18—C17	−2.5 (6)
C1—C2—C7—I1	4.0 (5)	C16—C17—C18—N3	1.3 (6)

### Hydrogen-bond geometry (Å, °)

**Table:**

*D*—H···*A*	*D*—H	H···*A*	*D*···*A*	*D*—H···*A*
O1—H1···O5^i^	0.84 (3)	1.64 (3)	2.478 (4)	174 (6)
O3—H3···O6^ii^	0.84 (3)	1.63 (3)	2.465 (4)	170 (7)
O1W—H1w1···O2	0.84 (3)	2.30 (3)	3.073 (4)	154 (4)
O1W—H1w2···O5^iii^	0.84 (3)	2.12 (3)	2.945 (4)	167 (5)
N1—H11···O1w^iv^	0.88 (3)	2.20 (3)	2.906 (5)	138 (4)

Symmetry codes: (i) −*x*+1/2, *y*−1/2, −*z*+3/2; (ii) −*x*+1, −*y*+1, −*z*+1; (iii) *x*+1/2, −*y*+3/2, *z*+1/2; (iv) −*x*+1/2, *y*+1/2, −*z*+3/2.
